# Fecal microbiota composition associates with the capacity of human peripheral blood monocytes to differentiate into immunogenic dendritic cells *in vitro*

**DOI:** 10.1080/19490976.2021.1921927

**Published:** 2021-05-10

**Authors:** Dušan Radojević, Sergej Tomić, Dušan Mihajlović, Maja Tolinački, Bojan Pavlović, Dragana Vučević, Svetlana Bojić, Nataša Golić, Miodrag Čolić, Jelena Đokić

**Affiliations:** a Laboratory for Molecular Microbiology (LMM), Institute of Molecular Genetics and Genetic Engineering (IMGGI), University of Belgrade, Belgrade, Serbia; b Department for Immunology and Immunoparasitology, Institute for the Application of Nuclear Energy, University of Belgrade, Belgrade, Serbia; c Faculty of Medicine Foca, University of East Sarajevo, Republic of Srpska, Bosnia and Herzegovina; d Medical Faculty of the Military Medical Academy, University of Defence, Belgrade, Serbia; e PHYTONET D.o.o, Belgrade, Serbia; f HITTest D.o.o, Belgrade, Serbia; g Serbian Academy of Sciences and Arts, Belgrade, Serbia

**Keywords:** Gut microbiota, SCFA, monocyte derived dendritic cells, anti-cancer vaccine, myeloid cell therapy

## Abstract

Although promising for active immunization in cancer patients, dendritic cells (DCs) vaccines generated *in vitro* display high inter-individual variability in their immunogenicity, which mostly limits their therapeutic efficacy. Gut microbiota composition is a key emerging factor affecting individuals’ immune responses, but it is unknown how it affects the variability of donors’ precursor cells to differentiate into immunogenic DCs *in vitro*. By analyzing gut microbiota composition in 14 healthy donors, along with the phenotype and cytokines production by monocyte-derived DCs, we found significant correlations between immunogenic properties of DC and microbiota composition. Namely, donors who had higher α-diversity of gut microbiota and higher abundance of short-chain fatty acid (SCFAs) and SCFA-producing bacteria in feces, displayed lower expression of CD1a on immature (im)DC and higher expression of ILT-3, costimulatory molecules (CD86, CD40) proinflammatory cytokines (TNF-α, IL-6, IL-8) and IL-12p70/IL-10 ratio, all of which correlated with their lower maturation potential and immunogenicity upon stimulation with LPS/IFNγ, a well-known Th1 polarizing cocktail. In contrast, imDCs generated from donors with lower α-diversity and higher abundance of *Bifidobacterium* and *Collinsella* in feces displayed higher CD1a expression and higher potential to up-regulate CD86 and CD40, increase TNF-α, IL-6, IL-8 production, and IL-12p70/IL-10 ratio upon stimulation. These results emphasize the important role of gut microbiota on the capacity of donor precursor cells to differentiate into immunogenic DCs suitable for cancer therapy, which could be harnessed for improving the actual and future DC-based cancer therapies.

## 1. Introduction

Cancer is a constantly rising world health problem and new improved approaches in anti-cancer therapy are desperately needed. Although many different therapies are being investigated, all of these have just a partial efficacy, thus the new therapy or combination of therapeutics are needed to increase the efficacy of cancer therapy. The suppression of immune response by cancer microenvironment was observed as the most important obstacle for successful cancer therapy. In addition to blockage of checkpoint inhibitors (CPIs) induction and stimulation of anti-cancer immune response by dendritic cells (DCs)-based vaccines are recognized as a very promising approach in cancer therapy.^1,2^ DCs represent a heterogeneous group of antigen-presenting cells (APCs), linking innate and adaptive immunity to provide an adequate immune response. DCs have a unique ability to uptake, process and express cancer antigens on their surface in the complex with the major histocompatibility complex (MHC) class I and II, to migrate to secondary lymph tissue where they induce activation of naïve cancer antigen-specific CD4 and CD8 T cells and their differentiation into effector cells. On the other hand, tolerogenic DCs which express high levels of tolerogenic molecules such as IL-10, Transforming Growth Factor (TGF)-β, Immunoglobulin-Like Transcript (ILT)3, ILT4, Programmed Death-Ligand (PD-L)1, Indoleamine 2,3-Dioxygenase (IDO)-1 display high capacity to induce differentiation of regulatory T cells (Tregs), and thereby suppress immune response.^3,4^ Properly activated DCs, expressing high levels of costimulatory molecules (i.e. CD86), CD83 and Th1-inducing cytokine IL-12, have the potential to trigger an efficient cancer-specific immune response mediated by cancer-antigen specific Th1 and cytotoxic T cells, which are armed with anti-cancer mechanisms.^5^ As the frequency of DCs in human peripheral blood is low,^6–9^ the protocols for *in vitro* differentiation of DCs from bone marrow precursor cells or peripheral blood monocytes have been explored extensively. Importantly, it was repeatedly noticed that there is a high donor-to-donor variability in DC precursors for *in vitro* differentiation of human peripheral blood monocytes into immunogenic DCs,^10^ which may lead to a large variability in the therapeutic efficacy of DCs.^11^ To date, the described sources of this variability are found in polymorphisms of genes coding cell receptors for maturation-inducing molecules such as Toll like receptors (TLRs) that recognize microorganisms-associated molecular patterns (MAMPs) and receptors for proinflammatory cytokines.^12,13^ However, it remained unclear whether additional factors are contributing to the variability of DCs generated *in vitro*.

A few years ago, Schirmer et al.^14^ pointed to the important correlation between the composition of gut microbiota and their metabolites and the inter-individual differences of healthy human donor immune cells to respond to different MAMPs. The human gastrointestinal microbiota consists of numerous bacteria, viruses, fungi, archaea, and protists which interact together and with the host, providing signals for immune response regulation, likewise, the immune system participates in the development and maintenance of the gut microbiota.^15^ The commensal bacteria provide immunomodulatory metabolites and nutrients such as short chain fatty acids (SCFAs), bile and amino acids, lipids, and vitamins.^16–18^ In addition to the differences among the healthy population in the gut microbiota composition, there is increasingly more evidence associating the abundance of specific gut bacteria and their metabolites, with immune-mediated diseases such as cancers and autoimmune diseases.^19^ Also, the efficacy of cancer immunotherapy based on immune CPIs have been correlated recently with the prevalence of the specific gut bacterial.^20–23^ However, it remained completely unknown whether gut microbiota composition and their metabolites may affect the phenotype and functional properties of *in vitro* generated DCs as a potential therapeutic modality in cancer immunotherapy. Therefore, the aim of our study was to identify specific gut microbiota members and their metabolites that correlate with the donor-related differences in phenotypic and functional markers expressed by human monocyte-derived DCs which point to their capacity to induce an effective anti-cancer immune response. Here we showed for the first time that gut microbiota composition, as well as fecal concentrations of SCFA, strongly predict for immunogenicity of DCs from different donors.

## 2. Results

### 2.1. The phenotype of monocyte-derived DCs and their response to stimulus vary highly between different donors

According to the three-signal model, the induction of effector T-cells by DCs requires the presentation of antigen/MHC, co-stimulatory signals, and polarizing cytokines.^24^ Immature DCs express a moderate level of MHC, but low levels of other signaling molecules such as CD86 and CD40. Upon stimulation with microbial pathogens or dangerous signals, DCs upregulate the expression of costimulatory molecules and cytokines enabling activation of naïve T cells and their differentiation into effector T cells.^25^ In line with this, many papers,^26^ including our own,^27^ showed that the stimulation of imDC with LPS/IFN-γ for 16 h provides an efficient maturation signal for DCs, potentiating their Th1 polarizing capacity.

**Figure 1.**
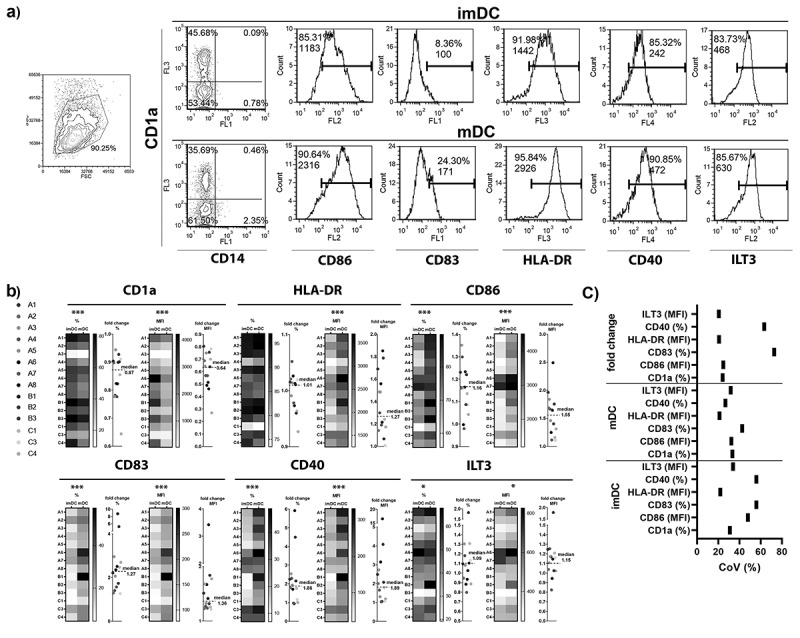
Interdonor variability of phenotypic markers expression on imDCs, and mDCs upon LPS/IFN-γ stimulation. (a) The gating strategy of DCs (FSC/SSC) and the phenotype analysis of one donor’s imDCs and mDCs are shown. The % of positive cells, and mean fluorescence intensity (MFI) of all gated DCs, are shown in each histogram. (b) Heatmaps represent markers expression on imDCs, and mDCs upon LPS/IFN-γ stimulation. The fold change of % or MFI for each phenotypic marker upon LPS/IFN-γ stimulation was calculated by dividing the level of marker expression on mDC by its level on imDC for each donor. The fold changes of marker expression upon LPS/IFN-γ stimulation are presented on dot plots for each donor in a different color. The phenotypic markers expression by imDC and mDC were compared by Wilcoxon matched-pairs signed-rank test. The statistically significant correlations are annotated with asterisks (***p < .001, *p < .05)

Therefore, to analyze donor-to-donor variability in the potential of human peripheral blood monocytes to differentiate into imDCs and mDCs we cultivated MACS-sorted monocytes in the presence of GM-CSF and IL-4 for 4 days in GMP medium, followed by their stimulation with LPS/IFN-γ (Figure 1.) Differentiation of monocytes toward imDCs is followed by a complete downregulation of CD14 and upregulation of CD1a.^28^ Flow cytometry analysis showed that monocytes from all donors (n = 14) indeed down-regulated CD14 expression during differentiation into imDC (min. 0.6% and max. 3.7% CD14^+^ cells). However, there was significant variability in the upregulation of CD1a expression on imDC between different donors. Upon stimulation with LPS/IFN-γ, CD1a expression on mDC was down-regulated on all donors, but a high variability in CD1a expression was observed on mDC as well. Moreover, variability in the fold change of CD1a down-regulation upon LPS/IFN-γ stimulation was observed. Similar to the expression of CD1a, other analyzed markers (CD86, CD83, HLA-DR, CD40) on imDCs and mDCs varied between the donors. Also, we noticed the variability of DCs in their capacity to respond on LPS/IFN-γ as fold change for all tested markers varied between the donors. In addition to markers related to immunostimulatory properties of DCs, we have analyzed the expression of ILT3, as a surface marker of tolerogenic DCs.^29^ The expression of ILT3 on imDCs varied between the donors, and similar variation in its expression was observed after LPS/IFN-γ stimulation. Interestingly, DCs from five donors did not change or slightly decreased the expression of ILT3 molecule upon LPS/IFN-γ stimulation, whereas DCs from nine donors upregulated its expression. There were no significant differences in the expression of analyzed markers between the group of eight donors tested in one-time point, compared to the group of six donors in the second time point (data not shown). Moreover, in a repeated experiment for one donor, the variability in expression of immune markers and in fold change upon the stimulation with LPS/IFN-γ, was quite low (Supplementary Figure 1). These results suggested that although the same protocol for differentiation and maturation of DCs was applied, there was a high variability in their phenotype and maturation capacity upon stimulation with LPS/IFN-γ.

### 2.2. The production of cytokines by monocyte-derived DCs varies highly between healthy donors

**Figure 2.**
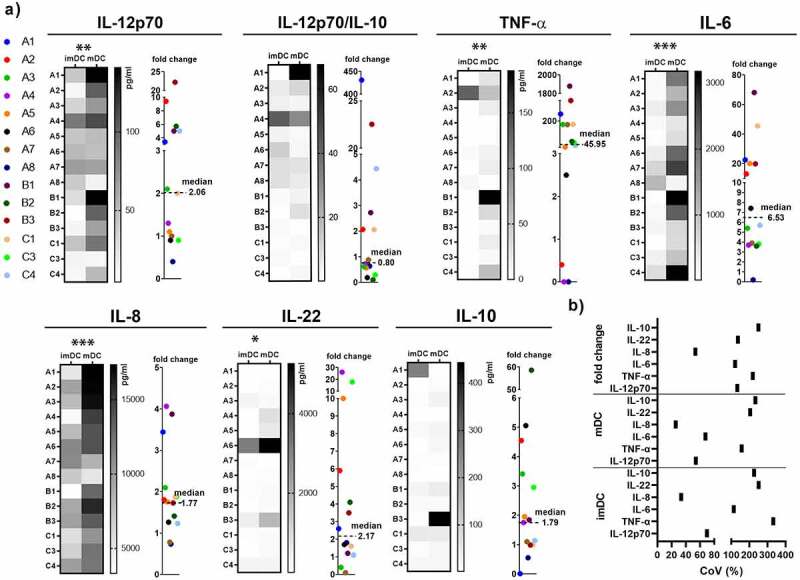
Interdonor variability of cytokine production by imDCs, and mDCs upon LPS/IFN-γ stimulation. Heatmaps represent cytokine production by imDCs, and mDCs upon LPS/IFN-γ stimulation. The fold change for each cytokine was calculated by dividing the level of cytokine produced by mDC, by the level of cytokine produced by imDC. The value of cytokine production fold change upon LPS/IFN-γ stimulation are presented on dot plots for each donor in a different color. The cytokines production by imDC and mDC were compared by Wilcoxon matched-pairs signed-rank test, by using GraphPad Prism 9.0. The statistically significant correlations are annotated with asterisks (***p < .001, **p < .005, *p < .05)

In addition to costimulatory molecules, the production of immunostimulatory, especially Th1 polarizing cytokines, is necessary for the induction of an efficient anti-cancer response. Accordingly, we have analyzed donor-to-donor variability in cytokines production by imDCs and mDCs upon stimulation with LPS/IFN-γ (Figure 2). We found that the capacity for the cytokines production by imDCs and mDCs varied between the donors even more than in phenotype (Figures 1c and 2b). The majority of DCs from healthy donors increased the production of IL-6 and IL-8 upon LPS/IFN-γ stimulation, and the exceptions were DCs from one donor which decreased the production of IL-6, and DCs from two donors which decreased the production of IL-8. DCs from nine donors responded to LPS/IFN-γ by elevating the levels of IL-12p70, four donors did not change its production (1 ± 0.1), whereas the production of IL-12p70 by DCs from one donor decreased upon stimulation. DCs from most donors elevated the production of IL-22 upon LPS/IFN-γ, whereas DCs from three donors did not significantly change the production of this cytokine. Besides immunostimulatory cytokines, we have analyzed immunosuppressive cytokine IL-10. Upon stimulation with LPS/IFN-γ, DCs from eight donors elevated the levels of IL-10, DCs from four donors did not change the expression and from two donors decreased the production of this cytokine. In addition to this, we also analyzed the ratio of IL-12p70/IL-10 levels as a measure of Th1 polarizing capacity of DCs. According to IL-12p70/IL-10 ratio, it appeared that DCs from only six donors elevated IL-12p70/IL-10 ratio upon LPS/IFN-γ stimulation. The variability in cytokines’ production by one DCs donor in a repeated experiment was low (Supplementary Figure 1).

### 2.3 The maturation capacity of monocyte-derived DCs negatively correlates with the expression of maturation markers on imDCs and the changes in ILT3 expression

**Figure 3.**
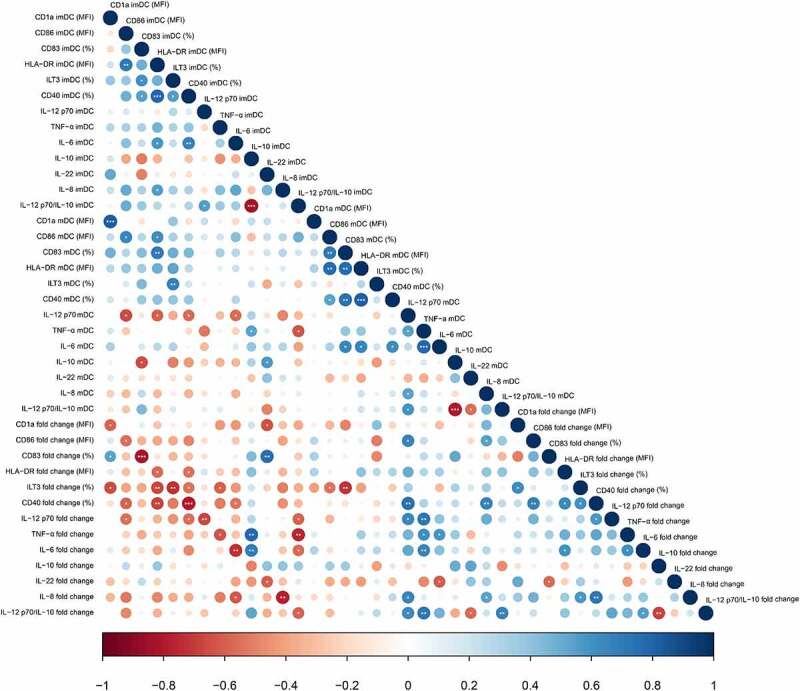
Spearman’s rank correlation between molecules expressed by imDCs and mDCs. The correlogram graphically represents the correlation between phenotypic markers and cytokines expressed by imDCs and mDCs, as well as fold change in molecules expression upon LPS/IFN-γ stimulation. Blue dots correspond to positive correlation and red dots to negative correlation. Dot size and color intensity are proportional to Spearman’s rho rank correlation coefficients. The statistically significant correlations are annotated with asterisks (***p < .001, **p < .01, *p < .05)

In order to perceive the relations between different molecules expressed by DCs, we investigated the underlying correlations between these molecules, taking into account their expression (% or MFI) on imDCs, mDCs and the levels of their change upon stimulation with LPS/IFN-γ (fold change) (Figure 3). The level of CD1a expression on imDCs positively correlated with the capacity of DCs to increase the levels of CD83 upon LPS/IFN-γ stimulation, and negatively correlated with their capacity to increase ILT3 upon the stimulation. Moreover, the expression of CD86 and CD83 on mDCs negatively correlated with the fold change in ILT3 expression. A positive correlation was observed between the expression of CD86, CD83, HLA-DR, CD40 and ILT3 on imDCs. However, the expression of all these markers on imDCs negatively correlated with the capacity of DCs to additionally increase their expression upon LPS/IFN-γ stimulation.

The production of IL-12p70, TNF-α, IL-6, IL-8, IL-22 as well as the IL-12p70/IL-10 production ratio by imDCs negatively correlated with the capacity of DCs to additionally increase (fold change) the production of these cytokines upon stimulation with LPS/IFN-γ. Also, IL-12p70/IL10 production ratio by imDCs negatively correlated with the fold change of this factor upon stimulation. The expression of CD86, HLA-DR, CD40, and IL-6 on imDCs negatively correlated with the level of IL-12p70 production by mDCs. The production of IL-12p70 on mDCs positively correlated with the capacity of DCs to increase CD86 and CD40 expression on LPS/IFN-γ stimulation as well as with the production of TNF-α and IL-8 by mDCs. The production of IL-10 by imDCs positively correlated with the capacity of DCs to increase TNF-α and IL-6 production upon stimulation with LPS/IFN-γ.

These results suggested that the phenotypic maturation and cytokines production by mDCs highly depend on variable phenotypic and functional status of imDCs from individual donors.

### 2.4 Variability in CD1a, TNF-α and IL-10 expression by monocyte-derived DCs associates with different gut microbiota diversity

**Figure 4.**
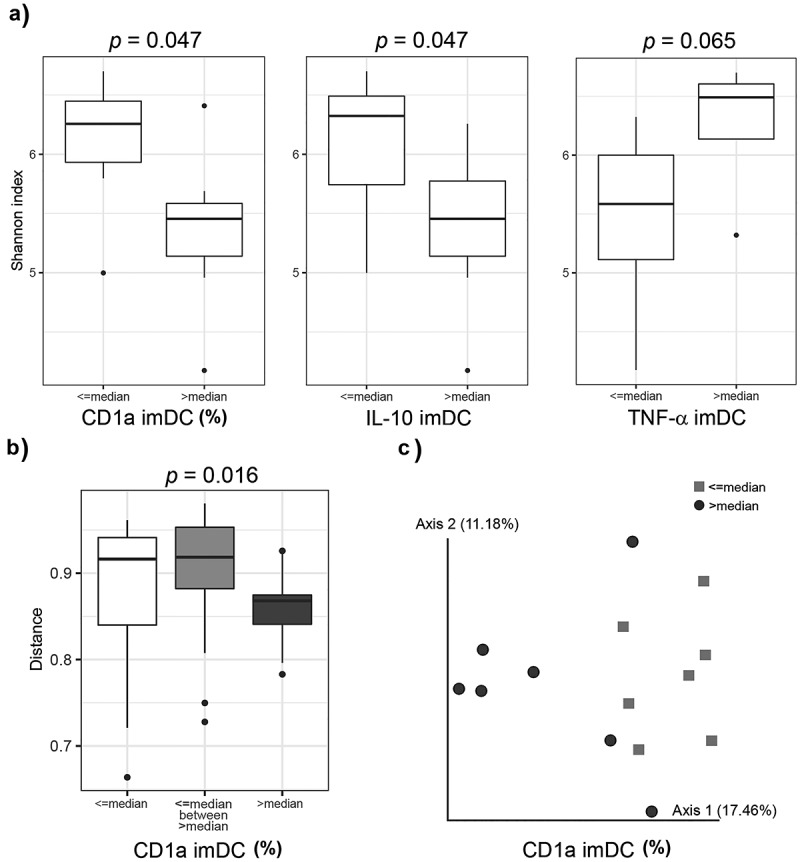
Differences in fecal microbiota diversity are associated with the capacity of healthy donors’ monocytes to differentiate into imDCs and mDCs. Box plots represent the comparison of Shannon diversity indices (a) between donors arranged in two groups according to each of the 60 immune markers expression at ≤ median and at > median level by DCs. Distance groups comparison between the donors that express the immune marker at ≤ median level by DC (white column), between the donors that express immune marker at ≤ median level by DC and the donors that express immune marker at > median level by DC (light gray column), and between the donors that express immune marker at > median level by DC (dark gray column) (b) are based on Bray-Curtis distance matrix and significance determined by ANOSIM following 999 permutations. Principal coordinates’ analysis plot (c) of beta diversity based on Bray-Curtis distance matrix for two groups of donors. Only statistically significant comparisons after correction with Benjamini-Hochberg procedure for multiple testing FDR are presented

It is recognized that immune response to pathogens varies across individuals and some have described this variability through common genetic variants,^30^ and others linked these variabilities to variations in microbiome composition.^14^ The variability in responsiveness of patients to drugs or immunotherapies also point to the association between responders/nonresponders and different parameters of gut microbiota.^31,32^ To investigate the potential role of microbiota in the capacity of monocytes to differentiate into imDCs, as well as their capacity to mature upon stimulation with LPS/IFN-γ, first we have analyzed the bacterial composition of fecal microbiota in healthy donors divided arbitrarily into groups based on the expression of immune molecules analyzed (Figure 4a). Additionally, donors were arranged in two groups for every analyzed immune marker according to its fold change upon LPS/IFN-γ stimulation (“≤ median” and “> median”). Thereby, significant differences were observed between the two groups of donors separated according to median values for all analyzed DC markers (data not shown).

By comparing the α-diversity, as a measure of donor’s gut microbiota diversity between two groups of donors divided according to the median of CD1a expression, we found that the donors whose imDCs expressed higher levels of CD1a (i.e. >median) had a significantly lower diversity of gut microbiota in comparison to donors whose imDCs expressed lower levels of this molecule. Further, imDCs from donors with significantly lower diversity of gut microbiota expressed lower levels of TNF-α. On the contrary, lower α-diversity was a characteristic of donors with higher levels of IL-10. There was no significant association between gut microbiota α-diversity and other analyzed markers of DCs.

Besides α-diversity, we tested whether donors whose imDCs express different phenotype and/or cytokine production or differentially responded to LPS/IFN-γ stimulation were similar based on β-diversity distances (Bray-Curtis distance), as a measure of gut microbiota compositional dissimilarity between the donors. There were significant differences in community composition between donors expressing different levels (≤ median and > median) of CD1a on imDCs (Figure 4b, 4c). Community composition between donors expressing lower levels of CD1a on imDCs was more diverse than between donors in the group expressing a higher level of this molecule on imDCs. There were no other significant differences in Bray-Curtis distances among groups arranged based on the expression of other molecules.

### 2.6 The variability in expression of CD1a, CD83, IL-12p70, TNF-α, IL-6, IL-10, and ILT3 associate with the abundance of different gut bacteria

**Figure 5.**
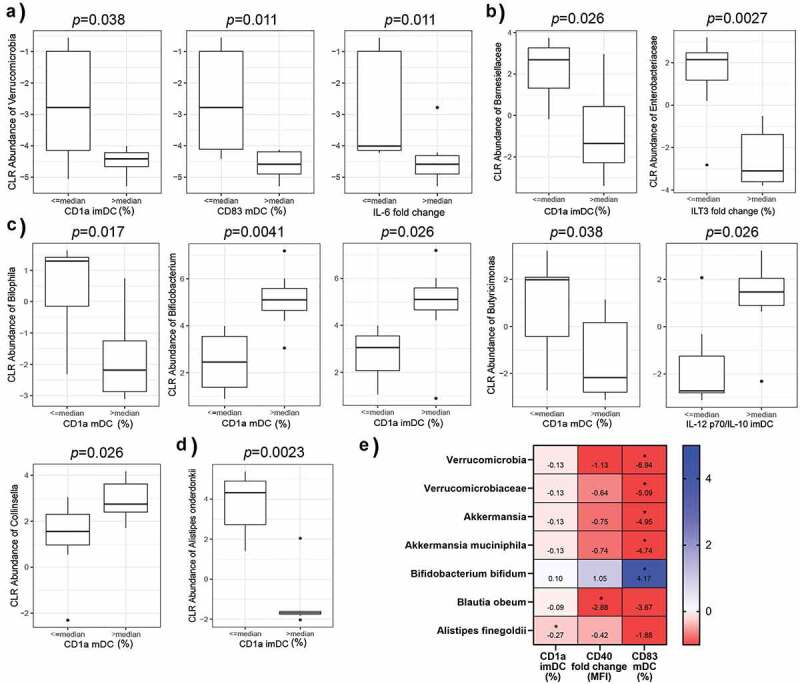
Different fecal microbiota composition is associated with the capacity of healthy donors’ monocytes to differentiate into imDCs and mDCs. Differential abundance of 6 phylum (a), 32 family (b), 58 genus (c), and 63 species (d) between two donors’ groups classified according to each of the 60 immune markers expression at ≤ median and at > median level by DCs. The analysis was performed by QIIME2 *p*-composition plugin for analysis of the composition of microbiomes (ANCOM) and all comparisons resulted in coefficient W higher than 1 statistical significance was confirmed with Pairwise Wilcoxon rank-sum test with the Benjamini-Hochberg procedure for multiple testing FDR control. To assess the relationship between the immune markers and bacterial taxa (same as A–D) linear regression analysis was performed (e) using R lm function, adjusting all models for age and gender. Beta coefficients and FDR adjusted *p* values were used for heat map generation in the Graph Pad software. For additional information see Materials and Methods. Only statistically significant comparisons after Benjamini-Hochberg procedure (FDR) are presented

As we showed that the diversity in immune response between the donors could be related to gut microbiota composition, we conducted differentially abundant taxa analysis at phylum, family, genus, and species level between the donor groups arranged in the same manner as the above correlation analyzes (Figure 5). In addition, we analyzed the association between the marker/cytokine expression and different bacterial taxa abundances by linear regression. First, donors with the higher expression of CD1a (> median) on imDCs or mDCs had a lower relative abundance of phylum Verrucomicrobia (Figure 5a), lower level of family *Barnesiellaceae* (Figure 5b), lower level of genus *Bilophila* and *Butyricimonas* (Figure 5c). The same donors contained higher levels of *Bifidobacterium* and *Collinsella* in feces and a lower level of species *Alistipes onderdonkii* (Figure 5d) than the donors whose CD1a expression on DCs was ≤ median. When analyzed by linear regression , the expression of CD1a on imDC was negatively associated with another species of genus *Alistipes, Alistipes finegoldi* (Figure 5e). In addition, a lower relative abundance of Verrucomicrobia, was associated with higher expression of the costimulatory molecule CD83 on mDCs and higher capacity of DCs to additionally increase the production of IL-6 on LPS/IFN-γ stimulation (Figure 5a). Also, in the analysis of association by linear regression, the expression of CD83 on mDC was negatively associated with the relative abundances of phylum Verrucomicrobia, family *Verrucomicrobiaceae* and species *Akkermansia muciniphila*, but was positively associated with the relative abundance of *Bifidobacterium bifidum*. Fold change of CD40 expression upon LPS/IFN-γ stimulation was negatively associated with the relative abundance of *Blautia obeum* (Figure 5e). On the other hand, donors whose DCs did not change or decreased the expression of ILT3 upon LPS/IFN-γ stimulation had a higher relative abundance of *Enterobacteriaceae* (Figure 5b). Interestingly, the differences between donors grouped according to the production of IL-12p70 solely did not differ significantly in their gut microbiota composition. However, the differences in microbial composition between the donors that differed in IL-12p70/IL-10 ratio were significant. Namely, we found that family *Odoribacteraceae* (with the genus *Butyricimonas*) is lower in the group of donors with the ratio of IL-12p70/IL-10 on imDCs ≤ median (Figure 5c).

### 2.7. Higher concentrations of fecal short chain fatty acids in donors negatively correlate with immunogenicity of their DCs generated *in vitro*

**Figure 6.**
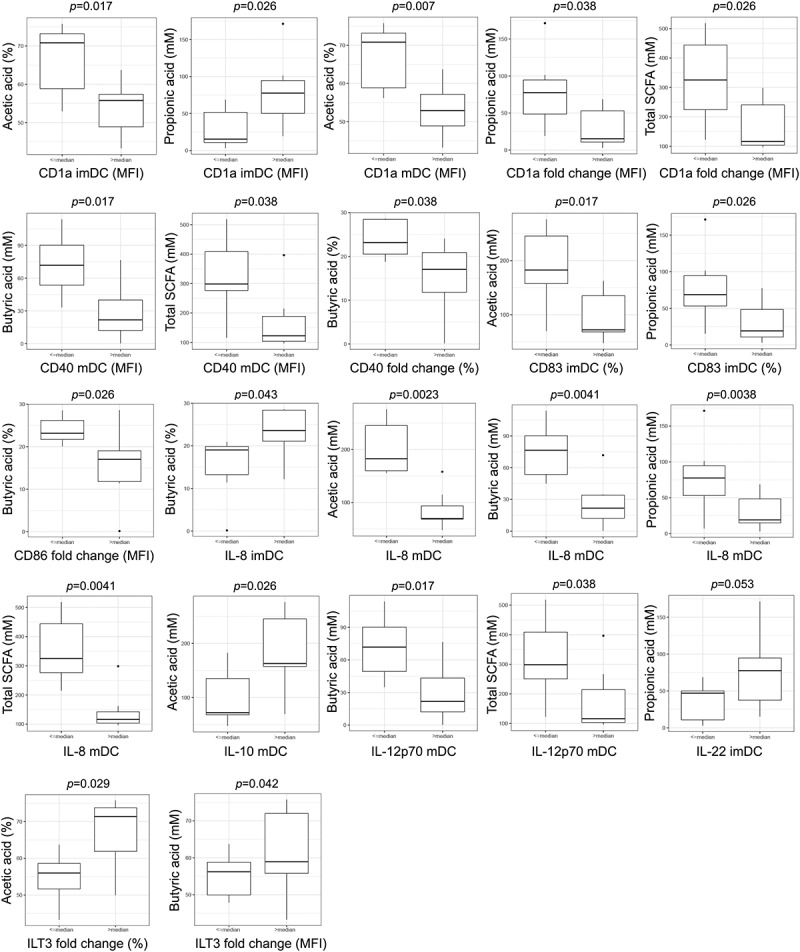
Association between fecal SCFA concentrations and donor-to-donor variability of differentiated DCs. Box plots represent statistically significant comparisons between two groups of donors arranged by immune markers expression level for fecal AA, PA, BA, and total SCFA concentrations (mM) and their relative contribution in the total SCFA. *p*-value for the Pairwise Wilcoxon rank-sum test less than 0.05 is considered significant

Short chain fatty acids (SCFA) are recognized as important products of bacteria in gut microbiota with a strong impact on various host functions. Among other effects, the immunomodulatory effects of these products are repeatedly described.^33^ Interestingly, by differentially abundance analysis, we found that higher abundance of genus *Butyricimonas* and species *Alistipes onderdonkii*, well-recognized SCFA producers,^34^ are found in donors whose DCs express lower levels of CD1a. Also, a higher abundance of genus *Butyricimonas* was found in donors whose imDCs display higher IL-12p70/IL-10 ratio and poorly increase IL-12p70/IL-10 production ratio upon stimulation with LPS/IFN-γ. To investigate further the potential association between fecal SCFA concentrations and donor-to-donor variability of differentiated DCs, we analyzed the concentration (mM) of total SCFA, acetic acid (AA), propionic acid (PA), and butyric acid (BA) as well as the relative contribution of each acid (%) in total SCFA (Figure 6). By the comparison of SCFA concentration/relative contribution between the donor groups arranged in the same manner as in the above analyzes, we found that the higher concentration of total SCFA or individual acids were present in donors whose DCs express lower levels of CD1a, but also lower levels of other molecules associated to immunostimulatory potential of DCs such as CD40, CD83, CD86, IL-8, and the most important Th-1-directing cytokine IL-12p70. On the contrary, we found that the higher fecal concentration of SCFA was characteristic of donors whose DCs express higher levels of immunosuppressive molecules IL-10 and ILT3.

## 3. Discussion

DCs generated *in vitro* from peripheral blood monocytes are a suitable source for cell therapy of cancers as a sufficient number of DCs can be obtained after differentiation of monocytes with GM-CSF and IL-4, followed by exposure to pro-inflammatory cytokines and/or TLR agonists.^35,36^ Extensive vaccination studies,^35–37^ demonstrated the capacity of DCs to prime cancer-specific T cells driving their differentiation into Th1 and CTL.^38–41^ In line with this, the high capacity of DCs to produce IL-12 and express high levels of co-stimulatory molecules (i.e. CD86, CD40) was shown critical for the induction of an efficient anti-cancer response.^41^ However, although immunogenic DC vaccines display clinical benefits for some patients, the majority of patients display poor or no clinical response to DC vaccines.^11^ Our results pointing to interdonor variability in phenotypical/functional characteristics of DCs differentiated from 14 healthy donors is in line with the variable clinical benefits of DC vaccines demonstrated in clinical studies. Considering the expression of the most important phenotypical markers, DCs obtained in our study differed greatly between donors at the stage of imDCs as well as after their exposure to LPS/IFN-γ. Although donor-to-donor variability has been observed for all analyzed markers in our study, the expression of markers important for T cell priming (HLA-DR, CD86, and CD40) increased in the majority of donors upon LPS/IFN-γ stimulation, and a few donors did not alter the expression of these markers. Interestingly, although ILT3 and IL-10 are known as the immunosuppressive molecules,^42^ DCs from the majority of donors upregulated their expression after LPS/IFN-γ stimulation. It is possible that the increment of ILT3 and IL-10 by DCs could be a result of negative feedback mechanisms in response to LPS/IFN-γ stimulation as the expression level of ILT3 on imDCs positively correlated with the expression level of CD86, CD83, and HLA-DR on imDCs. However, the increment of ILT3 upon LPS/IFN-γ stimulation negatively correlated with the potential of DCs to additionally increase the expression of CD86 and CD83. Considering these results, high ILT3 on imDCs could be a useful marker pointing to weak immunogenicity of DCs upon stimulation. In line with this, Chang et al. demonstrated that upregulation of ILT3 antagonizes the activation of DCs by TLR agonist,^43^ which could explain the negative correlation between ILT3 expression and maturation potential of DCs. In addition to membrane-bound molecules, the variability between donors in the production of important cytokines by DC has been shown to strongly impact the clinical efficacy of the vaccine.^44^ Subbiah et al.^44^ showed in phase I clinical trial significant correlation between the level of IL-12 and IL-8 production by DC and its efficacy in cancer therapy, pointing to the association between donor-to-donor variability in cytokines production and the immunogenicity of DC vaccine. Our results are in accordance with this phenomenon, as DCs from some donors even displayed decreased production of IL-12p70, TNF-α, IL-6, and IL-8 upon LPS/IFN-γ stimulation. In addition to the variability in the expression of proinflammatory cytokines, the production of IL-10 by LPS/IFN-γ stimulated DCs varied as well. Here we demonstrated that the IL-12p70/IL-10 ratio is a better marker for the identification of immunogenic DC than the usage of IL-12p70 as a sole marker. The identification of reliable markers of DC immunogenicity is of critical importance for the increased efficiency of DC-based cancer therapy. However, large variability in DC properties may hamper the efficacy of DC therapy for all patients. Lee et al.^30^ associated the inter-individual variance in a large set of genes with variability between donors’ DCs to respond to different microbial stimuli. On the other hand, the study by Mireia Uribe-Herranz et al.^45^ on cancer model in mice showed that the success of adoptive anti-cancer T cells transfer, as cancer therapy, is associated with the presence of different groups of bacteria in mice gut microbiome. These authors showed that the oral application of antibiotic contributed to the decrease in cancer growth which correlated with an increased number of IL-12 producing CD8α DCs. Also, different studies showed promising results on the role of gut microbiota modification in check-point blockade cancer therapy.^23,46^

The immunological parameters analyzed in this study as a measure of DC functions in healthy donors, are also relevant for the clinical efficacy of DC vaccines in cancer therapy. Previous studies, describing the variations in immune responses of healthy donors and patients, classified them commonly as responders and non-responders according to different values.^10,32,47,48^ This was the reason for separating the healthy donors in our study as weaker and stronger responders, besides using the linear regression for the analyses. Classifying donors into groups based on the DC markers, the Response Evaluation Criteria in Solid Tumors^48^ along with the microbiota analysis, could be very useful for identifying best predictive markers for successful therapy of cancer. Here we showed for the first time that the generation of immunogenic DCs *in vitro* significantly correlates with the gut microbiota composition, as demonstrated via analyses of α- and β- diversity and the abundance of specific taxa in relation to immune markers on DCs. Namely, we found that DCs from donors with the lower α-diversity of gut microbiota display better immunogenic parameters *in vitro*. Lower microbiota diversity correlated with a lower capacity of imDCs to produce TNF-α and a higher capacity to produce immunoregulatory IL-10, which could be associated with a non-activated state of the precursor immune cells in these donors. In accordance with these results, imDCs from the group of donors with lower α-diversity also displayed higher expression of CD1a, a marker shown to be strongly associated with a greater pro-inflammatory potential of DCs.^49^ The donors with the higher expression of CD1a had a more similar diversity of gut microbiota mutually (β-diversity), which could point to the fewer modifications in their microbiota composition and their less activated immune precursors. These results point to the previously unappreciated notion that donors with lower diversity of gut microbiota could be better candidates for anti-cancer DC therapy. This hypothesis could be supported by other studies,^45^ including our own,^50^ showing the association between the treatment of animals with oral antibiotics and their higher capacity to respond on immunostimulant. Therefore, antibiotic treatments of patients before sampling of their monocytes for autologous DC vaccine could be a promising approach to reduce the diversity of their gut microbiota and thereby increase the potential of their immune precursors to differentiate into immunogenic DCs.

Besides diversity in the microbiota, we also investigated if any of the detected bacterial taxa could be associated with the variability of DCs immunogenic phenotype and functions. The lower abundance of phylum Verrucomicrobia in gut microbiota was a characteristic of donors whose imDCs expressed higher levels of CD1a and expressed higher levels of CD83 and IL-6 upon LPS/IFN-γ stimulation. The only known member of this phylum in human gut microbiota is the mucolytic species *Akkermansia muciniphila*, which was described recently as the most important member of human gut microbiota contributing to mucus turn-over and gut barrier integrity.^51^ This species was shown to be beneficial for anti-PD-1 therapy, correlating with IFN-γ production by peripheral T cells and a more effective anti-cancer immune response^22^. Also, *Akkermansia muciniphila* produces both propionate and acetate, SCFAs with potential immunoregulatory properties. In that sense, the lower levels of Verrucomicrobia, and *Akkermansia muciniphila* in our study could point to the more nonactivated state of monocytes which could differentiate to imDCs with higher CD1a expression and stronger response on LPS/IFN-γ stimulation. Also, the relative abundance of *Akkermansia muciniphila* was negatively associated with the expression of CD83 on mDC pointing further to the immunoregulatory role of this bacteria. Schrimer et al.^14^ showed previously that the presence of *Barnesiella* in the gut microbiota of healthy donors negatively correlated with the capacity of their PBMC to produce IFN-γ in response to LPS stimulation. These results suggest that the presence of *Barnesiellaceae* in gut microbiota suppresses donors’ immune cells to respond to stimuli. In line with this, we found in the original model system that donors with a lower presence of *Barnesiellaceae* in gut microbiota contain monocytes that differentiate into imDC with higher levels of CD1a, and thereby mDC with increased immunogenicity. Interestingly, the study of Frankel et al.^52^ showed that *Barnesiellaceae* are more abundant in patients who do not develop adverse colitis upon receiving CTLA-4 check-point blockage cancer therapy, suggesting that the presence of this family correlates with a pronounced immunoregulatory phenotype of immune cells in these patients. This phenomenon could explain our original finding that the presence of genus *Bilophyla* was lower in donors whose DCs expressed higher levels of CD1a. Schirmer et al.^14^ showed that PBMC from donors with lower levels of *Bilophyla* in microbiota produces a higher level of TNF-α in response to LPS. Cumulatively, our study pointed for the first time that lower levels of Verrucomicrobia, *Barnesiellaceae*, and genus *Bilophyla* in gut microbiota increase the capacity of peripheral blood monocytes to differentiate into immunogenic DC *in vitro*. In addition to these most likely immunoregulatory bacterial taxa, the lower levels of *Butyricimonas* and *Alistipes onderdonkii* were found in donors whose DCs express a higher level of CD1a. Also, the relative abundance of *Alistipes finegoldii* was negatively associated with the expression of CD1a on imDC. Although different taxa were identified by different analysis approaches (linear regression and ANCOM analysis), both *Butyricimonas* and two species of *Alistipes* are well-known producers of SCFAs.^34,53^ Therefore, it is possible that the higher presence of SCFAs in these donors suppressed the potential of monocytes to differentiate into immunogenic DCs. Immunoregulatory properties of SCFAs in monocytes activation and DCs immunogenicity *in vitro* have been demonstrated previously,^54^ but this is the first time to show that the presence of SCFA-producing taxa and the presence of SCFAs in feces of healthy donors, negatively correlate with the potential of isolated peripheral blood monocytes to differentiate into immunogenic DCs *in vitro*. The molecular mechanisms of this phenomenon are still unknown and deserve further investigation. The lower presence of *Butyricimonas* was associated with the lower level of IL-12p70/IL-10 production by imDCs, which is a characteristic of imDCs with the higher capacity to respond to LPS/IFN-γ stimulation. Additionally, we demonstrated the association between SCFAs concentrations in feces with the reduced immunogenic characteristics of DCs. Namely, the concentration of total SCFAs and the relative contribution of each acid were significantly higher in the fecal material of donors whose DCs expressed lower levels of proinflammatory markers CD1a, CD40, CD86, CD83, and cytokines IL-8 and IL-12p70, but higher level of immunosuppressive IL-10 and ILT3. These results are in accordance with the already described protocols in which butyric acid impairs the differentiation of DCs from monocytes, their maturation *in vitro*,^54^ as well as their capacity to polarize naive CD4^+^ T cells toward IL-10-producing type 1 regulatory T cells.^55^ Various gut bacteria produce SCFAs as a major end-products of dietary fibers fermentation.^56^ Whereas most of butyrate is being utilized by colonocytes,^57^ the rest is being transported to the liver where most of acetate and propionate are being metabolized.^58^ Only a small part of microbial-derived SCFAs was shown to circulate in the blood. SCFAs contribute to the intestinal barrier function and exert direct immunomodulatory effects on intestinal epithelial cells contributing to the regulation of the immune system.^59^ The SCFAs measured in our study in the fecal material of donors, could exert the immunomodulatory effects by modulating the properties of intestinal epithelial cells that could affect the properties of circulating immune cells such as the monocytes. In order to test whether the higher level of SCFAs suppress the activation of different immune blood cells, including monocytes, the level of serum SCFAs has to be measured in future studies. As the properties of DCs to induce Th1 differentiation of naïve cancer-specific T lymphocytes and CTL is considered a prerequisite for a successful anti-cancer DC vaccine, the levels of butyric acid associated with the lower capacity of DCs to produce IL-12p70 in our study, could be the most significant therapeutic target. Even more warning, the higher concentration of this acid could be associated with the properties of differentiated DC to act immunoregulatory, as we found that DCs obtained from donors with higher levels of fecal butyric acid express higher levels of ILT3. Therefore, it can be postulated that microbiota modifications toward decreasing the abundance of SCFAs-producers in gut microbiota could be beneficial for cancer therapy and a good way for the preparation of the patients for DC vaccine. For this approach it is significant that SCFAs-producing bacteria were shown to be highly susceptible to diet modification, being supported by the fiber-rich diet, so the modification of patients’ diet in the mean of lower fiber food consumption in the period before monocytes sampling could be beneficial.

Most importantly, we showed for the first time in this study that genus *Bifidobacterium* and *Collinsella* were more abundant in donors whose DCs displayed pronounced immunostimulatory properties desirable for anti-cancer therapy. Namely, species of *Bifidobacterium* are known as a very potent probiotic or postbiotic fractions, which were shown to be effective in different immune-related diseases.^60^ This result opens the opportunity to investigate *Bifidobacterium* as a potential supplement to anti-cancer DC vaccine therapy. Strains of *B. animals* and *B. longum* that were shown to promote Th1 immune response could be good candidates for these investigations.^61–63^ In addition to live pro-inflammatory *Bifidobacterium*, the immunostimulatory components from these bacteria have been described, such as protein structure pili expressed by *B. bifidum*,^64^ as well as carbohydrates such as exopolysaccharides (EPS) produced by *B. breve* UCC2003.^65^ This is particularly important as we showed the positive association of *B. bifidum* with the expression of CD83 on mDC. As the immune system of cancer patients is already disturbed, the usage of isolated and fully characterized immunostimulatory postbiotics could represent a safe approach to restore the immunogenic potential of patients’ immune cells. We also showed that the higher presence of *Enterobacteriace* negatively correlates with the capacity of DC to increase ILT3 on LPS/IFN-γ stimulation. Some well described probiotic strains from this family, such as *Escherichia coli* Nissle 1917, could be a candidate for investigation on potential microbiota modification in order to obtain DCs that express lower levels of ILT3 upon LPS/IFN-γ stimulation.

In conclusion, our results are first connecting the variability between healthy donors in their microbiota composition with the variability in immunogenicity of their monocytes-derived DCs generated *in vitro*. This study pointed to the bacterial taxa such as genus *Bifidobacterium, Collinsella* and family *Enterobacteriaceae* that could be used as a supplemental therapy, and SCFA-producing bacteria that could be decreased by diet modification, both in order to increase the efficacy of anti-cancer DC vaccine. However, extending the study on a larger number of donors, especially cancer patients, is necessary to confirm this in future studies.

## 4. Materials and methods

### 4.1. Samples collection

Peripheral blood and fecal samples were collected from 14 donors (including 6 male and 8 female donors) without underlying immune system disorders and with blood test parameters in the reference range. All nonsmoker donors, with a median age of 33 (range 21–46), have not used antibiotics nor supplements in the previous six months and had not undergone an appendectomy. All donors gave voluntary informed consent to participate in experimental research, in accordance with the Ethical Board of Institute for Application of Nuclear Energy, University of Belgrade, No. 02–765/2 and Declaration of Helsinki. The peripheral blood samples and fecal samples of all donors (n = 14) were collected on the same day, allowing the association analysis between the immune parameters and the microbiota composition. Immediately after collecting, fecal samples were frozen at −80°C, whereas monocytes for DC differentiation were further isolated from peripheral blood samples.

PBMCs were obtained from buffy coats of healthy volunteers by using density gradient centrifugation on lymphocyte separation medium 1077 (PAA, Linz, Austria). Monocytes were separated from PBMCs by Magnetic activated cell sorting (MACS) as non-labeled fraction by using Pan monocytes isolation kit (Miltenyi Biotec, Bergisch Gladbach, Germany), and the purity of cells, according to flow cytometry analysis of CD14+ cells was higher than 90%. The cells were cultivated in CellGenix GMP DC medium supplemented with 100 ng/mL of human recombinant GM-CSF (Leucomax) and 20 ng/mL of human recombinant IL-4 (R&D Systems, Minneapolis, MN, USA) for 4 days to generate imDCs. imDC were treated for 16 h with 200 ng/ml of LPS from *Escherichia coli* 0.111: B4 (Sigma-Aldrich Co.) and 20 ng/mL of IFN-γ (R&D Systems, Minneapolis, MN, USA), in order to generate mDCs. To reduce possible technical errors during the cultivation and analyses, all DC cultures were carried out in totally two time periods. During one time period, eight (A donors) or six (B and C donors) DC cultures, were carried out, and later analyzed, simultaneously. Additionally, one donor provided blood samples twice within the three weeks to evaluate the reproducibility of DCs generation protocol in a repeated experiment.

### 4.2. Flow cytometry

DCs phenotype analysis was performed by flow cytometry (Partec Cube 6, Sysmex Partec GmbH, Germany) and BS LSR II (Beckton Dickenson) after labeling the cells with fluorochrome-conjugated primary antibodies in PBS/0.1% NaN3/0.5% FBS. In the analysis following antibodies (clone) were used: anti-CD14-FITC (TUK4), anti-CD83 biotin (HB15) (Miltenyi Biotec), anti-CD1a-PerCP/Cy5.5 (HI149), anti-CD83-FITC (HB15), anti-ILT-3-Pecy7 (ZM4.1), anti-HLA-DR-APCCy7 (L243), anti-CD86-PerCP/Cy5.5 (BU63),and anti-CD40-APC (all from Biolegend, San Diego, CA, USA), anti-CD86-PE (IT2.2), anti-ILT3-PE (ZM4.1) (all from Thermo Fisher Scientific, Waltham, MA, USA), and anti-HLA-DR-PerCP (L243) (R&D Systems, Minneapolis, MN, USA). The surface staining with primary Abs was conducted in PBS/0.1% NaN3/0.5% FBS. The signal overlap between the channels was compensated before each analysis using single labeled samples. Nonspecific fluorescence was determined according to fluorescence minus one (FMO) controls and isotype control antibodies, and at least 5000 cells were analyzed in each sample. Dead cells were gated-out according to 7-amino-actinomycin D (7AAD) staining, fixable viability dye 620 (BD) staining, or low FSC properties.

The cytokine levels produced by imDCs and mDCs (IL-12p70, IL1-β, TNF-α, TNF-β, IL-6, IL-10, IL-22, IL-8) were measured in cell-free supernatants by immunobead assay using LEGENDPlex system, according to manufacturer’s instructions in duplicates. The levels of IL1-β in the supernatant of DC cultures from 12/14 donors and TNF-β in 14/14 donors were below the detection limit of each cytokine, so these cytokines were not analyzed further.

The fold change of % or MFI for each phenotypic marker and cytokine upon LPS/IFN-γ stimulation was calculated by dividing the values of marker expression or cytokine production by mDC by the values for imDC for each donor.

### 4.3. SCFAs measurement

Fecal SCFAs extraction was performed following De Baere et al.,^66^ protocol. SCFAs concentrations were measured using high-performance liquid chromatography UltiMate 3000 UHPLC system (HPLC-UV) (Thermo Scientific, Breda, The Netherlands) with external calibration standards curve method as previously described in details.^67^

Briefly, calibration standards were prepared at concentrations ranging from 0.5 mM to 50 mM for acetic acid (AA), butyric acid (BA), propionic acid (PA), and succinic acid (SA) as internal standard (all purchased from Sigma-Aldrich, St. Louis, MO, USA). After chromatographic separation testing on a Hypersil Gold aQ column (150 × 4.6 mm i.d.) with a 3 μm particle (Thermo Scientific, Breda, The Netherlands), HPLC-UV was performed on thermostated and guard column protected HPLC columns using UV detection at 210 nm. The mobile phase consisted of 20 mM of sodium dihydrogen phosphate (Merck, Darmstadt, Germany) in HPLC water (pH 2.2) (Merck) (A) and HPLC grade acetonitrile (Sigma-Aldrich, St. Louis, MO, USA) (B).

HPLC-UV data were processed using Chromeleon version 6.8 software (Thermo Fisher Scientific, MA, USA). SCFAs concentration were calculated using mathematical equation: SCFA (AA, BA, PA) = (organic acid in fecal sample × 6 × 10^−3^)/(succinic acid in fecal sample × fecal sample mass) × 1000 [mmol/kg]. All measurements were done in triplicate.

### 4.4. Fecal DNA extraction and sequencing

Metagenomic DNA extraction from 14 frozen fecal samples was performed with ZR Fecal DNA MiniPrep™ Kit (Zymo Research Corp., Irvine, CA USA), according to manufacturer’s instruction in the sterile conditions (BSL2 level). Isolated DNA from all samples was stored at −20°C after PicoGreen DNA concentration measurements on Qubit™ fluorometer (ThermoFisher/Invitrogen, Waltham, MA USA). All samples were diluted to the concentration of 5 ng/μl in 10μl final volume and used for 16s rRNA amplicon sequencing, targeting V3-V4 hypervariable region. Paired-end sequencing was performed on MiSeq-Illumina platform at the FISABIO Sequencing and Bioinformatics Service (Valencia, Spain) via Science Exchange.

### 4.5. Statistical analysis and sequencing data processing

The phenotypic markers expression and cytokines production by imDC and mDC were compared by Wilcoxon matched-pairs signed-rank test, by using GraphPad Prism 9.0. The coefficient of variability used as a measure of variability in molecule expression between donors was calculated in GraphPad Prism 9.0 for each marker. To investigate the association between microbiota features and DCs’ properties we calculated the median value of expression for each immune marker and used median to separate the donors to weaker responders (expressing the marker less than or equal to the median value, “≤ median”) and better responders (expressing the marker greater than median value, “> median”). The differences between the donors separated according to this criteria for each marker were analyzed in RStudio v1.2.5042 (R Studio team) using Wilcoxon Rank Sum Tests with default parameters (paired = FALSE). Quality control of fecal microbial community sequencing data were assessed using a prinseq-lite program,^68^ with the following parameters: “min_length: 50, trim_qual_right: 30, trim_qual_type: mean, trim_qual_window: 20”. High-quality forward and reverse reads were joined using FLASH program,^69^ with default parameters. Bioinformatics platform QIIME2 v2020.2 (https://qiime2.org/)^70^ was used for additional filtering based on joined sequence quality scores, taxonomy assignment, and diversity measurement. Joined sequences imported into QIIME2 were processed with q-score-joined plugin using default parameters,^71^ and with deblur denoise-16S plugin,^72^ for the generation of denoised feature table and representative sequences based on *p*-trim-length 439. Taxonomy assignment was performed using the feature classifier trained using the Greengenes 13_8 99% OTUs,^73^ and samples rarefied at sampling depth 4000 were used for further diversity analyses. The quantitative measure of community richness and evenness (Shannon’s index) was calculated between the donors classified in the groups based on immune marker expression at ≤median, and at >median level by DCs, for all the analyzed phenotypic markers and cytokines. The community dissimilarity (Bray-Curtis distance) was observed using the beta-group-significance plugin with *p*-method “anosim,” following 999 permutations for determining the differences between the groups.^74^ Significant differences in β-diversity between the groups were visualized using EMPeror principal coordinates analysis (PCoA) biplots.^75,76^ Diversity boxplots were created using ggplot2^77^ package in RStudio v1.2.5042, and Pairwise Wilcoxon rank-sum test, with the Benjamini-Hochberg procedure for multiple testing false discovery rate (FDR) control, was used for comparing the Shannon indices between the donor groups. Differential taxa abundance analysis between the donor groups in all analyzed variables was performed by QIIME2 *p*-composition plugin,^78^ for analysis of the composition of microbiomes (ANCOM) at different taxa levels (60 immune markers vs 6 phyla, 32 families, 58 genera, and 63 species). For all the comparisons with the W coefficient higher than 1, the statistical significance was confirmed with Pairwise Wilcoxon rank-sum test with the Benjamini-Hochberg procedure for multiple testing FDR control and plotted in RStudio using several packages, qiime2R,^79^ phyloseq,^80^ microbiome,^81^ ggplot2,^77^ and stats (R Core Team). To additionally assess the relationship between the immune markers and bacterial taxa, the linear regression analysis was performed by using R lm function, adjusting all models for age and gender. The pseudocount addition (constant value of 0.0001) and log transformation were applied when the non-normal distribution of residuals occurred. Beta coefficients and FDR adjusted *p* values were used for heat map generation in the Graph Pad software. SCFA concentrations (mM of total SCFA, mM concentration, and relative contribution of acetic, propionic, and butyric acids) were compared between the donor groups for all the immune parameters applying the Pairwise Wilcoxon rank-sum test, and the results were visualized using ggplot function. R packages Hmisc,^82^ and corrplot^83^ were used for Spearman’s rank correlation matrix generation and visualization. The data for this study have been deposited in the European Nucleotide Archive (ENA) (https://www.ebi.ac.uk/ena) under the accession number PRJEB41873 and the secondary accession number ERP125715.

## Supplementary Material

Supplemental Material
