# Supplementary figures and images for: Fecal microbiota composition associates with the capacity of human peripheral blood monocytes to differentiate into immunogenic dendritic cells in vitro
Source: Gut Microbes. 2021 May 10;13(1):1921927. doi: 10.1080/19490976.2021.1921927 (PMC8115579; doi:10.1080/19490976.2021.1921927)

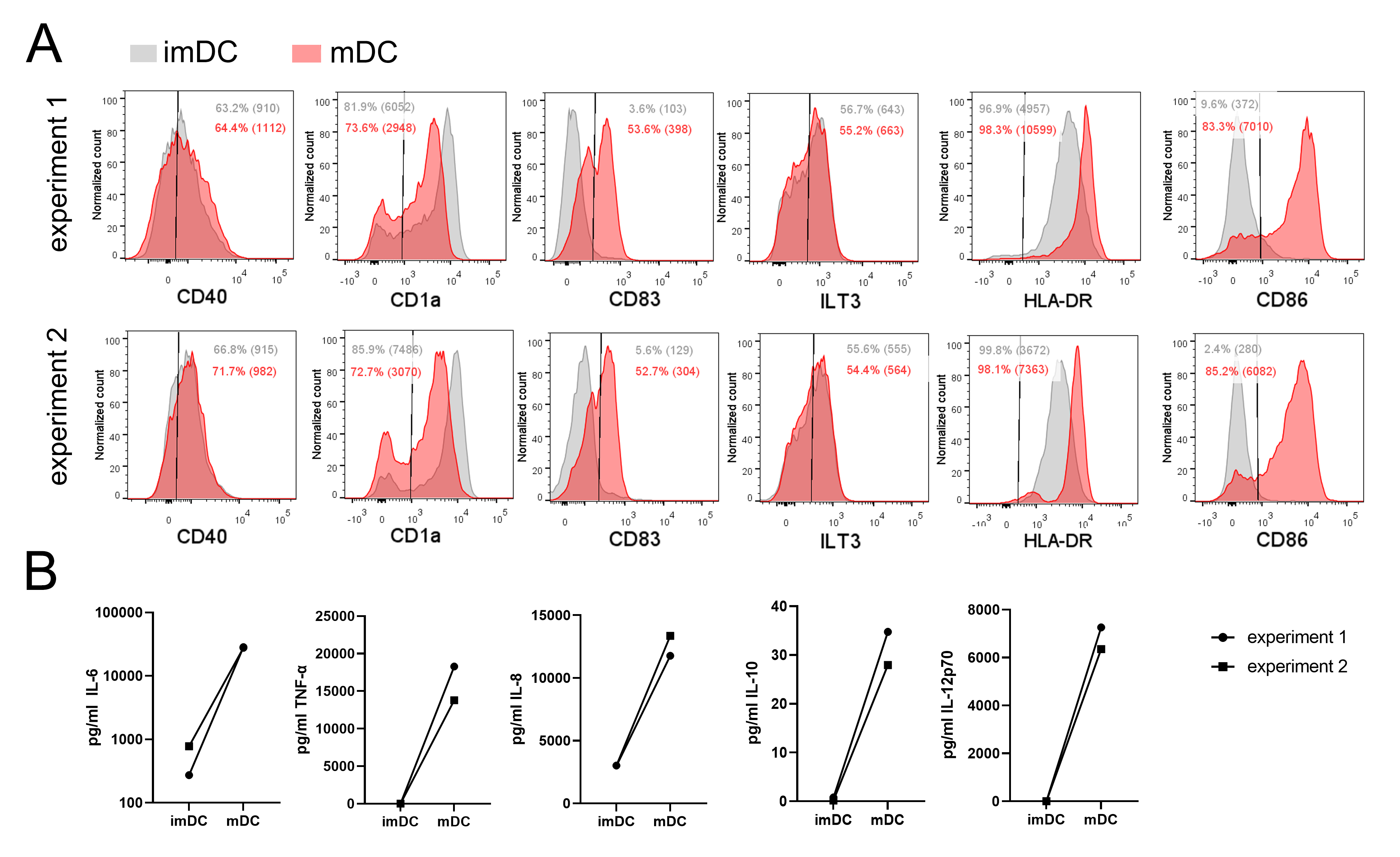

Supplement: Supplemental Material [file KGMI_A_1921927_SM0610.tif]
